# The silence of loneliness: echoes of loss from my cancer patients

**DOI:** 10.1093/oncolo/oyae366

**Published:** 2024-12-31

**Authors:** Stanislav Lazarev

**Affiliations:** Department of Radiation Oncology, Icahn School of Medicine at Mount Sinai, New York, NY 10029, United States

As a radiation oncologist, I have the privilege of accompanying patients and their families through some of the toughest chapters of their lives. Over the years, I have developed deep connections with many of my patients, their loved ones, and the stories they share with me. However, these experiences often come with an overwhelming sense of loss and loneliness—feelings that are rarely discussed among those in my profession.

One patient, in particular, had a lasting impact on me. She was an amazing woman—a teacher, a wife, a mother, and an incredibly kind, warm, and gracious human being. I had treated her for liver cancer and had come to know her and her family—her daughters and husband—over several years since our first meeting in the clinic. We shared moments of optimism and relief as she made remarkable progress during her treatment. Her cancer had been stable for so long that I never anticipated the call I would receive just a couple of weeks after a routine follow-up, when she had shared her plans for the summer. It was her husband who called me, letting me know that she had suddenly fallen ill over the weekend. Within just 24 hours, she had passed away, taken by a sudden late complication of her liver cancer.

I was stunned. While talking to her husband, I struggled to contain my emotions, feeling a wave of sorrow that I could barely hold back. What struck me was that despite his own grief, he expressed how much his wife had valued my care, sharing how my words and actions had helped her feel at ease during times of uncertainty, especially when her cancer recurred. He shared that, in many ways, her perseverance and determination to keep going, even when her cancer journey looked grim, were shaped by the kindness and support she found in our clinic during her treatment. Yet, as I sat alone in my office later that day, I was overwhelmed by sadness and found myself in tears.

This wasn’t the first time I had lost a patient, and it won’t be the last. As a doctor who treats cancer—often in advanced and incurable stages—I have walked this path many times, as have many of my colleagues. Yet, each experience is unique and deeply painful, and we each process this pain differently. What lingers is not just the grief but also a profound sense of isolation.

You want to share your feelings with someone, to express how heavy this loss weighs on you, but finding an outlet can be challenging. Your colleagues, as compassionate as they may be, often have their own patients and concerns. You hesitate to burden them. At home, you grapple with the decision to bring this sadness into your family’s life. As a result, the grief remains contained, swirling in a quiet space that offers no easy release.

This loneliness is not simply a result of frequent encounters with death; it arises from a deeper structural and emotional isolation within the field. The unspoken expectation of resilience often discourages sharing grief with colleagues, while the demands of patient care and administrative responsibilities leave little room for self-reflection. As cancer physicians, we experience a unique form of professional loneliness, one that is exacerbated by the repetitive nature of loss and limited opportunities to process emotions. Although support groups and mentorship could offer solace, such resources are often scarce or overlooked, leaving many of us to cope alone. Over time, this solitude can weigh heavily.

Another patient who left a lasting impact on me was a young woman with brain cancer, whom I treated a few years ago, and who was also a doctor. Her diagnosis had come just a year after she had given birth, leading to a challenging journey against a grim prognosis. I had known her for so long, having seen her for follow-ups almost every month, that her case became one of the most profound connections I have had in my career. She faced her illness with resilience that I rarely see in others. Over the 2 and a half years I knew her, we spoke not only about her treatment but also about life. She knew when I was still dating my wife, when I proposed, when I got married, and, more recently, when my wife became pregnant. While other patients had shared insights about cherishing life, it was through her journey that I truly grasped the depth of those lessons. She taught me to appreciate every day we are alive and to value every aspect of our physical being. She encouraged gratitude for the present moment, reminding me that tomorrow is never guaranteed and that we should embrace life here and now—every small moment of it: waking up in the morning, seeing the smile of a neighbor, and feeling the hug of a loved one.

During one of our last conversations, she told me she didn’t think she’d live to see our next appointment. Hearing those words struck me deeply. I struggled to hold back tears when, all of a sudden, she asked, “When is your baby due?” I choked up, realizing that while I was preparing to welcome new life, she was preparing to say goodbye. The contrast was heartbreaking. She ended that conversation by telling me she hoped we would meet again, whether in this life or in the next. Her last words to me were a poignant reminder: when my son is born, I should hug him every day and cherish every moment, because each moment with him would be golden. It was a reminder of the preciousness of life, a sentiment that carried even more weight knowing she was a mother herself.

Then, just a few days ago, I received a call from the father of a patient I had treated several years ago for metastatic breast cancer—a wonderful, vibrant young woman, a chef who could connect with anyone in a matter of seconds and was always full of life—who had passed away nine months earlier. She had been much younger than me, and over the course of her treatment, we developed a warm, trusting connection. I had watched her do well, and seen her cancer go into remission and return several times. I knew her struggles intimately. She had been there from the time I started as an attending physician after residency, and as I grew in my role, she was one of the patients who knew me, my story, and the key moments of my personal and professional life.

Her father shared his experiences of the months following her passing and the profound grief he still felt. Hearing him recount his journey of coping with the loss, reflecting on what he had shared with his daughter while she was alive, and describing how he still talks to her when he visits her grave was both cathartic and heart-wrenching. It brought forth the grief I had felt at her loss, reminding me of the emotional weight we carry as doctors—not only for our patients but for their families as well. It was a stark reminder of how deeply our patients’ stories stay with us, even months or years after they’ve gone.

After the call, I found myself once again grappling with a profound sense of loneliness. Sitting at my desk, I recalled the moments I had shared with his daughter as her physician and replayed our conversations in the clinic. This connection felt especially meaningful because of the strong bond we had built over years of shared struggles, small victories, and difficult conversations. Unlike personal experiences with death, these moments with patients carry a unique weight—they are deeply tied to our professional roles as physicians, yet evoke a profoundly personal grief. The grief and loss we experience as oncologists often don’t end when a patient’s life does; they linger for months or even years.

In medicine, no amount of experience makes it easier to confront death. While we may grow in our clinical skills and deepen our understanding of the disease, the loneliness that accompanies the loss of patients never fades. It is a quiet, constant companion in our profession, one that many of us carry without ever truly discussing it. Yet, in those rare moments when we connect with patients’ families, we find solace—a reminder that even in grief, we are not entirely alone. These shared moments become a quiet but powerful source of comfort, helping us carry on with our work and process the unspoken weight we all bear.

